# Congener-Specific Dietary Exposure and Predicted Organ-Specific Toxicity of Halogenated PAHs in Populations Living near a Coking Industrial Area

**DOI:** 10.3390/jox16030079

**Published:** 2026-05-05

**Authors:** Yanpeng Gao, Weijie Lu, Yibo Zhang, Mingze Geng, Xianglong Luo, Yuemeng Ji, Yang-Guang Gu

**Affiliations:** 1 Guangdong-Hong Kong-Macao Joint Laboratory for Contaminants Exposure and Health, Guangdong Key Laboratory of Environmental Catalysis and Health Risk Control, Institute of Environmental Health and Pollution Control, Guangdong University of Technology, Guangzhou 510006, China; gaoyp2016@gdut.edu.cn (Y.G.); 13650615863@163.com (W.L.); 3123005518@mail2.gdut.edu.cn (Y.Z.); 13753833198@163.com (M.G.); jiym@gdut.edu.cn (Y.J.); 2 Guangdong Basic Research Center of Excellence for Ecological Security and Green Development, Key Laboratory of City Cluster Environmental Safety and Green Development of the Ministry of Education, School of Environmental Science and Engineering, Guangdong University of Technology, Guangzhou 510006, China; 3 South China Sea Fisheries Research Institute, Chinese Academy of Fishery Sciences, Guangzhou 510300, China

**Keywords:** halogenated polycyclic aromatic hydrocarbons (HPAHs), dietary exposure, coking industry, food contamination, toxic equivalency factors (TEFs)

## Abstract

Halogenated polycyclic aromatic hydrocarbons (HPAHs) are persistent contaminants with elevated toxicity, yet dietary exposure data remain limited. Here, we systematically assessed dietary HPAHs using 87 duplicate diet samples collected from populations living in and around a coking industrial area by applying the duplicate diet method, a gold-standard approach that provides precise individual-level exposure information. Thirty-one HPAHs were detected, including seven previously unreported congeners, with mean concentrations of 62.51, 33.68, and 16.52 ng/g in the coking plant, nearby residential, and control areas, respectively. Lipid-rich foods, particularly meats, exhibited the highest HPAH burdens, and thermal processing approximately doubled concentrations in meals collected from the coking plant area. Dietary cancer risk was evaluated using a toxicity equivalency-based incremental lifetime cancer risk (ILCR) framework. Although several HPAHs occurred at low concentrations, congeners with high relative effect potency contributed disproportionately to cumulative cancer risk. Population-level risk distributions revealed that 25.8% of dietary samples exceeded the benchmark ILCR threshold of 10^−4^ in the coking plant area. In silico toxicity predictions further indicated potential organ-specific toxicological relevance for the blood, liver, kidney, and cardiovascular systems, supporting the health relevance of dietary HPAH exposure. In general, these results suggest that industrial influence, food composition, and cooking practices jointly contribute to dietary HPAH exposure and toxicity-weighted cancer risk. Our findings highlight the importance of incorporating halogenated congeners into routine monitoring programs and health risk assessments in industrialized regions.

## 1. Introduction

Halogenated polycyclic aromatic hydrocarbons (HPAHs), formed through the substitution of hydrogen atoms in parent PAHs by halogen elements such as chlorine or bromine, have recently emerged as a class of contaminants of increasing environmental and toxicological concern [1,2,3]. Compared with their parent PAHs, HPAHs generally exhibit higher chemical stability, stronger lipophilicity, and enhanced resistance to environmental degradation, which may promote their persistence in food matrices and biological tissues [3,4,5]. Toxicological evidence further suggests that certain HPAHs possess dioxin-like activities, endocrine-disrupting potential, and carcinogenicity that may equal or exceed those of unsubstituted PAHs [6,7,8]. Despite these concerns, HPAHs remain largely absent from routine environmental monitoring programs and food safety regulations, resulting in substantial uncertainty regarding their exposure pathways and associated health risks.

Dietary intake has been recognized as one of the dominant exposure routes for hydrophobic organic contaminants in the general population [9,10,11]. For conventional PAHs, numerous studies have demonstrated that food consumption—particularly of meat, cereals, vegetables, and processed foods—contributes more substantially to human exposure than inhalation or dermal contact (e.g., [11,12,13,14,15]). In contrast, knowledge regarding dietary exposure to HPAHs remains fragmented. Existing studies have primarily focused on the occurrence of HPAHs in environmental matrices such as atmospheric particles, soils, sediments, and industrial emissions (e.g., [2,5,8]), while systematic investigations targeting foodstuffs are still scarce. Moreover, most available dietary studies are limited to a small number of compounds or food categories, hampering a comprehensive understanding of exposure profiles across different dietary habits. As a result, the translation of environmental HPAH contamination into actual human dietary exposure remains poorly understood, representing a critical bottleneck in evaluating their real-world health relevance.

Industrial activities involving high-temperature combustion and chlorination or bromination processes are considered major anthropogenic sources of HPAHs [2,8,16]. Among these, the coking industry has been identified as a particularly important contributor due to the combined effects of coal pyrolysis, halogen-containing additives, and complex emission pathways [1,2,17]. Previous research has documented elevated concentrations of parent PAHs in environmental media and agricultural products surrounding coking facilities, suggesting a heightened exposure risk for nearby populations [18,19,20]. However, whether similar spatial patterns apply to HPAHs, and to what extent coking-related emissions influence population-level dietary exposure in surrounding communities, remain poorly characterized. Population-based assessments of dietary exposure to HPAHs in industrially impacted regions are extremely limited, representing a critical gap in current environmental health research. Addressing this gap requires integrating food-borne HPAH contamination with population-oriented dietary exposure frameworks that explicitly consider industrial emission contexts.

Another major challenge in assessing HPAH-related health risks lies in the pronounced structural diversity and congener-specific toxicity of these compounds. Different halogen substitution patterns and ring structures can result in substantial variability in bioaccumulation potential and toxic potency [2,21,22]. While toxic equivalency factors (TEFs) have been proposed for a limited number of HPAHs, their application in dietary risk assessment remains rare. Consequently, most previous studies have relied on concentration-based evaluations without adequately addressing differences in toxicological relevance among individual congeners. This limitation may lead to significant under- or overestimation of actual health risks associated with dietary exposure. A congener-resolved risk perspective is therefore essential for identifying risk-dominant HPAHs and for avoiding misleading conclusions based solely on total concentrations.

Food processing and preparation practices further complicate the assessment of dietary HPAHs [23,24,25]. Thermal treatment, curing, and seasoning processes have been shown to influence the formation and accumulation of parent PAHs in foods [12,23,24,25,26], yet their effects on halogenated analogues are poorly understood. In addition, environmental contamination during cultivation, livestock feeding, and atmospheric deposition may play a critical role in determining HPAH levels in raw and processed foods [2,4,27]. Distinguishing the relative contributions of environmental background contamination and food processing–related inputs is therefore essential for identifying effective risk mitigation strategies, but has rarely been addressed for HPAHs. Integrating food-type characteristics, processing practices, and environmental source influences remains an underdeveloped but necessary step in dietary HPAH exposure assessment.

Against this background, a comprehensive dietary exposure assessment of HPAHs in industrially influenced regions is urgently needed. Such an assessment should integrate a wide spectrum of food categories representative of local consumption patterns, incorporate congener-specific analysis of HPAHs because differences in halogen substitution pattern and molecular structure can substantially affect environmental behavior and toxic potency, and apply health risk metrics that account for differences in toxic potency. This is particularly important because total-concentration-based evaluation may obscure the contribution of low-abundance but highly potent congeners to overall dietary risk. Importantly, comparisons among populations with varying degrees of industrial influence can provide valuable insights into the role of emission sources in shaping dietary exposure profiles.

In this study, we develop an integrated framework to characterize dietary exposure to HPAHs and evaluate associated health risks among populations residing in and around a typical coking industrial area. By combining extensive food sampling across multiple categories with congener-resolved chemical analysis and toxic equivalency-based risk assessment, this work aims to address key knowledge gaps regarding the occurrence, exposure pathways, and potential health implications of HPAHs in human diets. By explicitly linking industrial emission contexts, food contamination profiles, and congener-specific health risk metrics within a population-based dietary exposure framework, this study provides new insight into how HPAHs are transferred from industrial sources into human diets. The findings are expected to support improved interpretation of HPAH behavior in food systems, contribute to more accurate dietary risk assessments, and provide a scientific basis for future monitoring and regulatory considerations targeting halogenated PAHs in industrial regions.

## 2. Materials and Methods

### 2.1. Study Area and Sample Collection

Shanxi Province is the largest coke-producing region in China, with an annual output of 98.6 million tons in 2021, accounting for approximately 21% of national coke production, highlighting the regional significance of coking-related emissions. The study was conducted in Lüliang City, Shanxi Province, northern China, a region characterized by a temperate semi-arid continental monsoon climate. Lüliang City represents a typical coal-based industrial region in northern China, with intensive coking activities accompanied by related industries such as steel production, coal-fired power generation, petroleum processing, and chemical manufacturing.

Industrial facilities are primarily concentrated in the southeastern part of the city, whereas the western and northern areas are dominated by residential communities and agricultural land, forming a clear spatial gradient of industrial influence. According to the most recent population census, the city has approximately 226,800 permanent residents, providing a representative population for assessing industrially influenced dietary exposure.

Based on differences in proximity to industrial activities, the study area was categorized into three zones: a coking plant area, an exposed residential area, and a control area. The approximate distances between the coking plant area and the exposed residential area and control area were ~1 km and ~50 km, respectively (Figure S1). To protect sensitive information related to industrial infrastructure, specific sampling coordinates are not disclosed, and only schematic maps are provided to illustrate relative spatial relationships among the three areas. Dietary samples were collected using the duplicate diet method, which involves collecting identical portions of all foods consumed by participants over a 24 h period. This method accounts for contamination introduced during food preparation, cooking, and processing, as well as food losses during consumption, thereby providing an accurate representation of actual dietary intake [11,28].

Participants were recruited from the three study zones using a field-based voluntary sampling approach. Eligible participants were adults who lived and/or worked in the corresponding study area and were willing to provide a complete 24 h duplicate diet sample together with questionnaire information on demographic characteristics, dietary habits, and cooking practices. Individuals with incomplete dietary records or insufficient sample collection were excluded from the study. All participant-based sampling procedures were conducted in accordance with relevant ethical guidelines, and informed consent was obtained from all participants prior to sample collection. The study protocol was reviewed and approved by the Medical Ethics Committee of Tongji Medical College, Huazhong University of Science and Technology.

To ensure representative sampling, structured questionnaires were administered to participants to obtain information on demographic characteristics, dietary habits, and cooking practices across the coking plant area, exposed residential area, and control area (Table S1). A total of 87 duplicate diet samples were collected (Table S2), including 31 samples from participants associated with the coking plant area, 18 samples from residents in the exposed residential area, and 38 samples from residents in the control area. The exposure grouping was defined primarily according to spatial proximity to the coking industry and the corresponding industrial influence gradient, rather than occupation alone. Specifically, the coking plant area represented a high-exposure scenario and included participants linked to the coking plant setting, whereas the exposed residential area and control area represented residential exposure scenarios with intermediate and low industrial influence, respectively. Thus, the present study was designed to compare real-world dietary exposure under different industrial influence contexts, while occupation and residence were not fully disentangled in the highest-exposure group. Immediately after collection, all dietary samples were sealed in clean low-density polyethylene (LDPE) food storage bags and placed in cooled containers with ice packs for transport to the laboratory. Upon arrival at the laboratory, all food samples were carefully weighed, homogenized, transferred into 250 mL amber glass bottles, and then stored at −20 °C until further processing and analysis. Samples were carefully handled, separated, and prepared individually to minimize the risk of cross-contamination throughout the sampling and analytical procedures.

### 2.2. Sample Preparation and Instrumental Analysis

The study employed the duplicate diet method, widely regarded as a “gold standard” for assessing dietary exposure due to its accuracy [11,28]. Although resource-intensive, this approach provides precise individual exposure data [11,29]. The standard solution containing 31 HPAHs and the fluorinated polycyclic aromatic hydrocarbon (F-PAH) standards were purchased from AccuStandard (New Haven, CT, USA). Ethyl acetate, hexane, acetonitrile, dichloromethane, and isooctane were all of high-performance liquid chromatography (HPLC) grade and were obtained from CNW Technologies GmbH (Düsseldorf, Germany). Dietary samples collected by this method comprised complex cooked food matrices containing multiple food items, condiments, and seasonings, with high lipid content and abundant pigments. Such complexity poses significant challenges for the determination of HPAHs, particularly in terms of efficient extraction and lipid removal. To address these challenges, an optimized sample preparation procedure was developed for reliable quantification of multiple HPAHs.

Briefly, 1.0 g of homogenized food sample was weighed into a 50 mL polypropylene centrifuge tube (Tube A). A mixture of isotopically labeled internal standards (Nap-d_8_, Ace-d_10_, Phe-d_10_, Chr-d_12_, and Per-d_12_) was added and allowed to equilibrate for 30 min. These deuterated parent-PAH standards were used as surrogate internal standards because a complete set of isotopically labeled H-PAH analogues was not commercially available. They were selected to span a broad range of molecular weights and chromatographic retention behavior, thereby providing correction across different classes of target H-PAHs. Their practical suitability for the present method was supported by the overall method-validation results, including acceptable recoveries, precision, and linearity (Table S3), although compound-specific differences among H-PAHs cannot be entirely excluded. Subsequently, 2 mL of ultrapure water and 5 mL of acetonitrile were added, followed by vortex mixing for 3 min and ultrasonic extraction for 10 min. The extract was stored at 4 °C for 2 h to facilitate phase separation. After cooling, 2 g anhydrous MgSO_4_ and 0.5 g NaCl (QuEChERS salt mixture) were added, vortexed for 1 min, and centrifuged at 10,000 rpm and 4 °C for 5 min.

The supernatant was transferred to a 15 mL centrifuge tube (Tube B) containing SupelTM QuE Z-Sep sorbent and anhydrous MgSO_4_ for enhanced lipid removal. After vortexing for 3 min and centrifugation at 10,000 rpm and 4 °C for 5 min, the resulting supernatant was transferred to a 10 mL centrifuge tube (Tube C). To improve analyte recovery, an additional 2 mL of acetonitrile was added to Tube B, and the extraction, vortexing, and centrifugation steps were repeated. Combined extracts were gently evaporated under nitrogen and reconstituted to a final volume of 1.5 mL in autosampler vials, to which 50 μL of fluorinated PAHs (F-PAHs, 50 ng/mL) was added as injection standards, corresponding to 2.5 ng per extract. Prepared samples were stored at −20 °C until analysis.

Quantitative determination of 31 HPAHs was performed using a Shimadzu GCMS-TQ8040 (Shimadzu Corporation, Kyoto, Japan) gas chromatograph–triple quadrupole mass spectrometer equipped with a DB-5MS capillary column (30 m × 0.25 mm × 0.25 μm). One microliter of each sample was injected in splitless mode at 280 °C. Helium (99.999%) was used as the carrier gas at 1.0 mL/min. The oven program was: 80 °C for 0.5 min, ramped to 280 °C at 5 °C/min, then to 300 °C at 15 °C/min (held 15 min), followed by a final ramp to 315 °C at 20 °C/min (held 0.5 min). The transfer line and ion source temperatures were 300 °C and 230 °C, respectively, with electron ionization (EI) and multiple reaction monitoring (MRM) used for target compound quantification.

### 2.3. Quality Assurance and Quality Control (QA/QC)

Method reliability was evaluated using procedural blanks, spiked samples, and replicate analyses. Method recoveries, calculated by subtracting concentrations in non-spiked samples from those in spiked samples, ranged from 79.24% to 107.46% for all HPAHs, indicating acceptable method accuracy (Table S3). The QA/QC parameters reported in Table S3 cover all target HPAHs analyzed in this study, including the seven previously unreported congeners identified in dietary samples. Precision, expressed as relative standard deviation (RSD, %), ranged from 0.68% to 10.94% across five replicate analyses of spiked samples, demonstrating good reproducibility (Table S3).

Limits of detection (LODs) and quantification (LOQs) were 0.0010–0.0478 ng/g and 0.0054–0.1222 ng/g, respectively (Table S3). Seven-point external calibration curves (5–500 ng/mL) exhibited excellent linearity for all compounds (R^2^ > 0.99), confirming reliable quantification. All solvents were of chromatographic grade (CNW Technologies), and ultrapure water was used throughout. Glassware was baked at 450 °C for 8 h prior to use to remove potential organic contaminants. Procedural blanks confirmed negligible background contamination during sample preparation and analysis.

### 2.4. Dietary Exposure and Health Risk Assessment

The potential carcinogenic risk associated with dietary exposure to HPAHs was assessed using the incremental lifetime cancer risk (ILCR) model recommended by the United States Environmental Protection Agency [30], which has been widely applied to organic contaminants such as PAHs, HPAHs, and polychlorinated biphenyls (PCBs). This approach integrates compound-specific toxicity with long-term dietary intake to estimate population-level cancer risk. Because duplicate diet samples represent the actual foods consumed within a 24 h period, the measured HPAH concentrations provide a direct estimate of short-term dietary intake on the sampling day. In the present study, these measured concentrations were further used as a screening-level proxy for longer-term dietary exposure under the assumption that the sampled meals broadly reflect habitual dietary patterns and local exposure contexts within each study group. Therefore, the ILCR values reported here should be interpreted as indicative estimates of chronic dietary cancer risk at the group level, rather than precise lifetime risk predictions for individual participants.

Given the pronounced congener-specific toxicity of HPAHs, carcinogenic risk was evaluated using a toxic equivalency–based framework. The toxic equivalency (TEQ_i_) of HPAHs relative to benzo[a]pyrene (BaP) was calculated according to Equation (1):

(1) $${TEQ}_{i}=\sum \left(C_{i}\times {REP}_{BaP,i}\right)$$

where $C_{i}$ represents the concentration of an individual HPAH congener in food, and ${REP}_{BaP,i}$ is its relative effect potency with respect to BaP. For interpretive purposes, the TEQ-derived BaP-equivalent dietary exposure term represents the toxicity-weighted dietary intake component used in the ILCR framework prior to application of the slope factor and lifetime adjustment terms. The ILCR associated with dietary exposure to HPAHs was then calculated using Equation (2):

(2) $$ILCR=\frac{\sum_{i=1}^{n}{TEQ}_{i}\times IR\times ED\times {SF}_{BaP}}{BW\times AT}$$

where IR is the dietary ingestion rate (kg d^−1^), ED is the exposure duration (years), ${SF}_{BaP}$ is the oral cancer slope factor of BaP (7.3 mg/kg/d), BW is body weight (kg), and AT is the averaging lifetime (years). Based on established risk criteria, ILCR values < 10^−6^, between 10^−6^ and 10^−4^, and >10^−4^ were interpreted as negligible, potential, and unacceptable cancer risks, respectively.

Parameter values were selected to represent typical adult exposure conditions in China. Dietary ingestion rates were set at 1.033 kg/d for rural residents and 1.1177 kg/day for urban residents, assuming daily food consumption throughout the year (365 days/year). The exposure duration was set to 45 years, corresponding to the average age of the study participants, while body weight and lifetime were assumed to be 64 kg and 70 years, respectively. These parameter settings represent a simplified adult exposure scenario and do not explicitly capture inter-individual variability in food intake, body size, or long-term exposure duration. Accordingly, they may influence the absolute magnitude of ILCR estimates, although they remain useful for comparative risk characterization across exposure groups.

Due to the limited availability of experimentally derived toxicological data for many HPAH congeners, relative effect potency factors (${REP}_{BaP}$) were derived using a combined literature-based and quantitative structure–activity relationship (QSAR) approach. Carcinogenicity and mutagenicity of HPAHs were first predicted using the OECD QSAR Toolbox (version 4.5), and reference compounds with high structural similarity (i.e., similar ring number, halogen substitution pattern, and molecular framework) were selected accordingly (Table S4). Subsequently, carcinogenic potency values (CPVs) for target HPAHs and their reference analogues were estimated using the CTV model. For HPAHs without available experimental REP data but with suitable structural analogues, ${REP}_{BaP,\;i}$ values for HPAHs without available experimental data were then calculated using Equation (3):

(3) $${REP}_{BaP,i}=\frac{{CPV}_{i}\times {REP}_{BaP,j}}{{CPV}_{j}}$$

where ${CPV}_{i}$ and ${CPV}_{j}$ represent the predicted carcinogenic potency values of the target HPAH and its reference analogue, respectively, and ${REP}_{BaP,j}$ is the known relative potency of the reference compound. The average prediction error of the CTV model is approximately 0.97 log_10_ units, and its applicability domain covers more than 80% of environmental organic chemicals. Using this approach, ${REP}_{BaP}$ values were obtained for 18 HPAH congeners, either directly from the literature or through structural extrapolation (Table S4). This combined strategy expands the congener coverage of cumulative risk assessment, but it also introduces uncertainty because QSAR-assisted REP_BaP estimates may deviate from the true biological potency of individual HPAHs, particularly for congeners lacking direct toxicological validation. Therefore, the resulting TEQ and ILCR values should be interpreted as screening-level, toxicity-weighted estimates that are suitable for comparative assessment, rather than exact toxicity-equivalent or lifetime cancer-risk values for specific individuals.

### 2.5. Data Analysis

Data analysis and visualization were performed using R software (version 4.4.3). For dietary exposure and health risk assessment, concentrations of HPAHs below the limit of detection (LOD) were treated as zero in the primary analysis as a lower-bound assumption. This approach was adopted because the duplicate diet samples were large composite food samples analyzed using optimized extraction and enrichment procedures, such that non-detects were considered more likely to reflect very low concentrations than clear analytical limitations. However, we acknowledge that alternative substitution approaches (e.g., LOD/2 or LOD) could lead to higher absolute estimates of HPAH concentration, TEQ, and ILCR, particularly for congeners with low detection frequencies.

ILCR values were calculated for individual dietary samples using a toxicity equivalency–based framework. To characterize population-level cancer risk patterns, ILCR values were log_10_-transformed to reduce right-skewness and facilitate comparison across exposure groups. Formal statistical comparisons of log_10_-transformed ILCR among the coking plant area, exposed residential area, and control area were performed using the Kruskal–Wallis test, followed by pairwise Wilcoxon rank-sum tests with Holm adjustment for multiple comparisons. These nonparametric methods were selected because ILCR data showed evident right-skewness and unequal dispersion among groups. Probability density distributions of log_10_-transformed ILCR were estimated using kernel density estimation (KDE) with a Gaussian kernel, providing a non-parametric representation of population-level cancer risk distributions, including central tendency, dispersion, and high-risk tails. Accordingly, comparisons among the coking plant area, exposed residential area, and control area were based on both formal nonparametric statistical testing and descriptive evaluation of distributional shifts, overlap, and tail behavior relative to established cancer risk benchmarks (10^−6^ and 10^−4^). Because only one duplicate diet sample was collected per participant, temporal variability in food consumption, cooking practices, and HPAH concentrations across days or seasons was not explicitly characterized. Accordingly, the exposure and ILCR estimates derived in this study should be regarded as snapshot-based measurements anchored to the sampling day, while their interpretation for chronic risk relies on the assumption that these samples broadly reflect typical local exposure conditions at the population level.

### 2.6. Sensitivity Analysis

To evaluate the robustness of the toxicity-weighted dietary cancer-risk estimates, a scenario-based sensitivity analysis was performed for the fixed exposure assumptions used in the ILCR model. Because ILCR is proportional to TEQ × IR × ED/BW, lower- and upper-bound exposure scenarios were generated by varying dietary ingestion rate (IR), body weight (BW), and exposure duration (ED) around the base-case assumptions. Specifically, the lower-bound scenario was defined as IR −20%, BW +20%, and ED = 30 years, whereas the upper-bound scenario was defined as IR +20%, BW −20%, and ED = 60 years. ILCR values were recalculated under each scenario, and the stability of both the absolute risk magnitude and the between-group risk pattern was evaluated across the coking plant area, exposed residential area, and control area.

## 3. Results

### 3.1. HPAH Detection Frequencies

**Figure 1:**
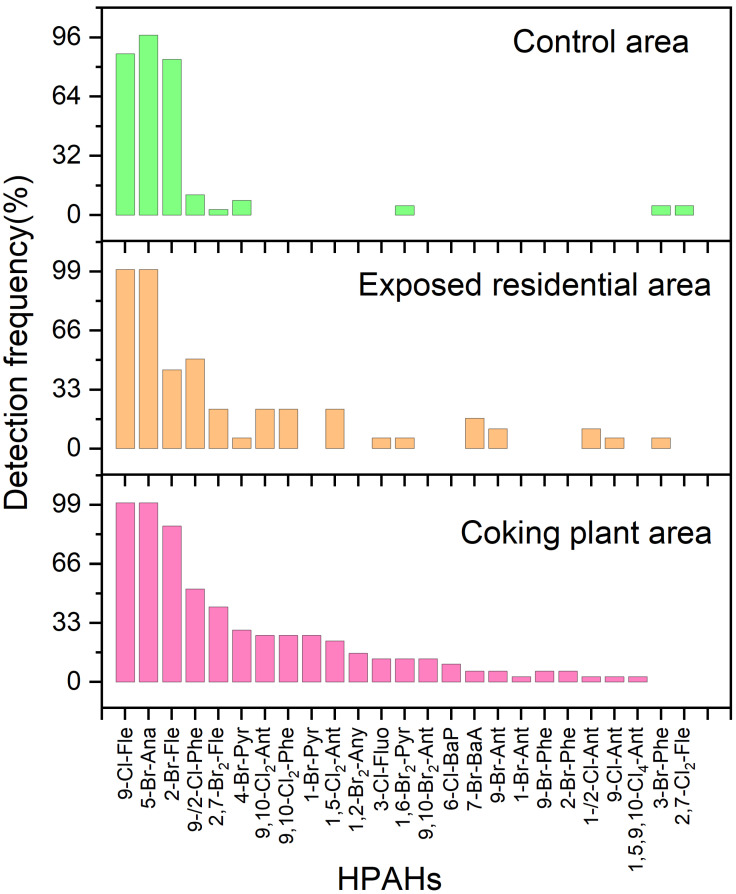
Detection frequencies of 31 halogenated polycyclic aromatic hydrocarbons (HPAHs) in dietary samples from coking plant worker, residents in exposed areas, and control area residents.

The detection rates of 31 HPAHs in dietary samples from coking plant workers, residents in exposed area, and control area residents are shown in Figure 1. Three HPAHs—5-Br-Ana (98.9%), 9-Cl-Fle (94.3%), and 2-Br-Fle (77.0%)—were detected in over 75% of samples (*n* = 87). Seven additional HPAHs, including 9-Cl-Phe, 2-Cl-Phe, 2,7-Br2-Fle, 4-Br-Pyr, 9,10-Cl2-Phe, 9,10-Cl2-Ant, and 1,5-Cl2-Ant, were found in 12.6–33.3% of samples. The remaining 22 HPAHs were detected in fewer than 10% of samples, including four that were undetected (1-Cl-Pyr, 3-Br-Fluo, 9,10-Br2-Phe, 7-Cl-BaA). A total of 25, 18, and 10 HPAHs were detected in the coking plant, exposed residential, and control areas, respectively. Notably, seven previously unreported HPAHs (1,5-Cl2-Ant, 1,5,9,10-Cl4-Ant, 5-Br-Ana, 1,2-Br2-Any, 2-Br-Phe, 4-Br-Pyr, and 9,10-Br2-Phe) were identified, most lacking existing toxicity equivalency factor (TEF) data, highlighting potential novel dietary contaminants.

### 3.2. HPAH Concentrations and TEQ Estimates

Mean concentrations of ∑31HPAHs were 62.51 ng/g in the coking plant area, 33.68 ng/g in the exposed residential area, and 16.52 ng/g in the control area (Table S5). The highest concentrations were observed for 9-/2-Cl-Phe in the coking plant area (8.24 ± 2.29 ng/g) and control area (4.69 ± 0.26 ng/g) and 1-/2-Cl-Ant in the exposed residential area (6.05 ± 0.73 ng/g).

Due to limited TEF data for many HPAHs, USEPA-based cancer risk assessments may underestimate exposure. Using the CTV model (14), TEQs were recalculated for HPAHs lacking TEFs (Table S5), allowing identification of moderate-to-high toxicity compounds such as 1,5-Cl2-Ant, 9,10-Br2-Ant, 9,10-Br2-Phe, and 1,5,9,10-Cl4-Ant. Corrected TEQs were 6.259 ng/g (coking plant), 2.456 ng/g (exposed residential), and 0.486 ng/g (control), with a >1.5-fold increase observed in the coking plant area. The TEQ-based BaP-equivalent dietary exposure level in the coking plant area was 2.6 times higher than that in the exposed residential area and five times higher than that in the control area. Lifetime average daily BaP exposure in coking plant workers was 2.6 times higher than in the exposed residential area and five times higher than in the control area.

### 3.3. HPAH Profiles in Canteen Meals

**Figure 2:**
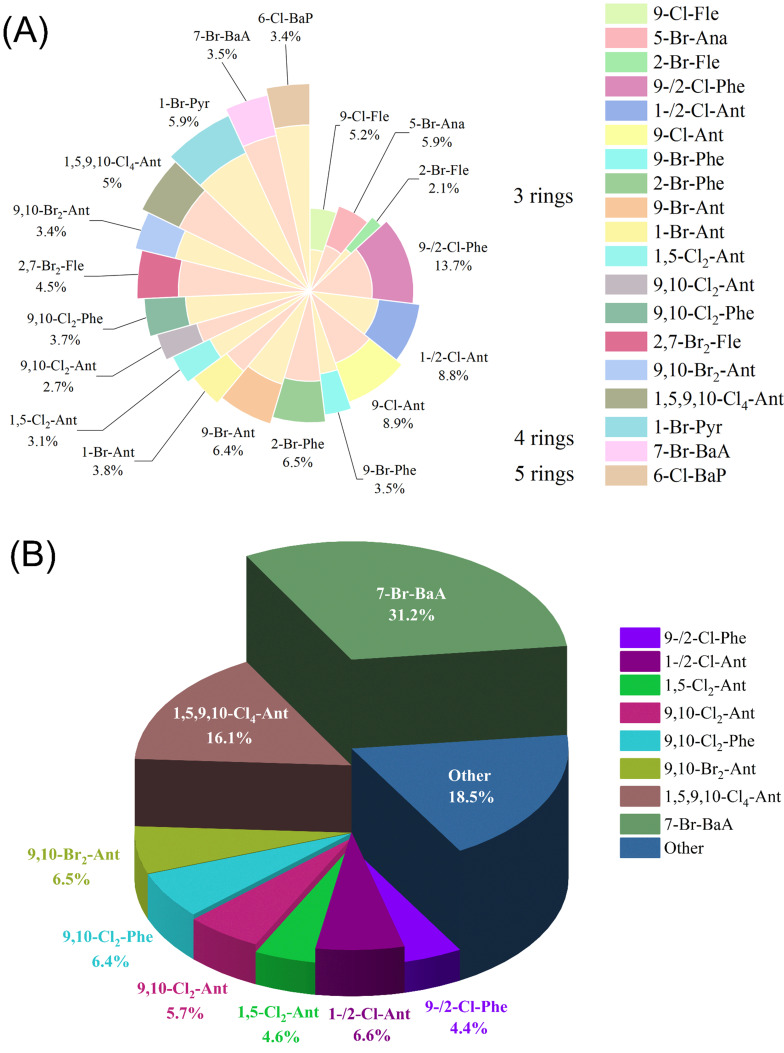
Concentration distributions of HPAHs (**A**) and their toxicity equivalents (TEQs) (**B**) in cafeteria meal samples from the coking plant.

Analysis of 21 HPAHs in cafeteria meals revealed 9-/2-Cl-Phe as the most abundant (13.7% of total HPAHs), followed by 9-Cl-Ant (8.9%) and 1-/2-Cl-Ant (8.8%) (Figure 2A). Although three-ring HPAHs dominated the concentration profile, several low-abundance congeners contributed disproportionately to toxicity; notably, the four-ring compound 7-Br-BaA accounted for less than 4% of total HPAHs but contributed 31.2% of total TEQ. TEQ analysis (Figure 2B) showed the highest contribution from 7-Br-BaA (31.2%), followed by 1,5,9,10-Cl4-Ant (16.1%) and 9,10-Br2-Ant (6.5%). Over 80% of total HPAHs in cafeteria meals were halogenated derivatives of Fle, Phe, and Ant.

### 3.4. HPAH Distribution Across Food Types

**Figure 3:**
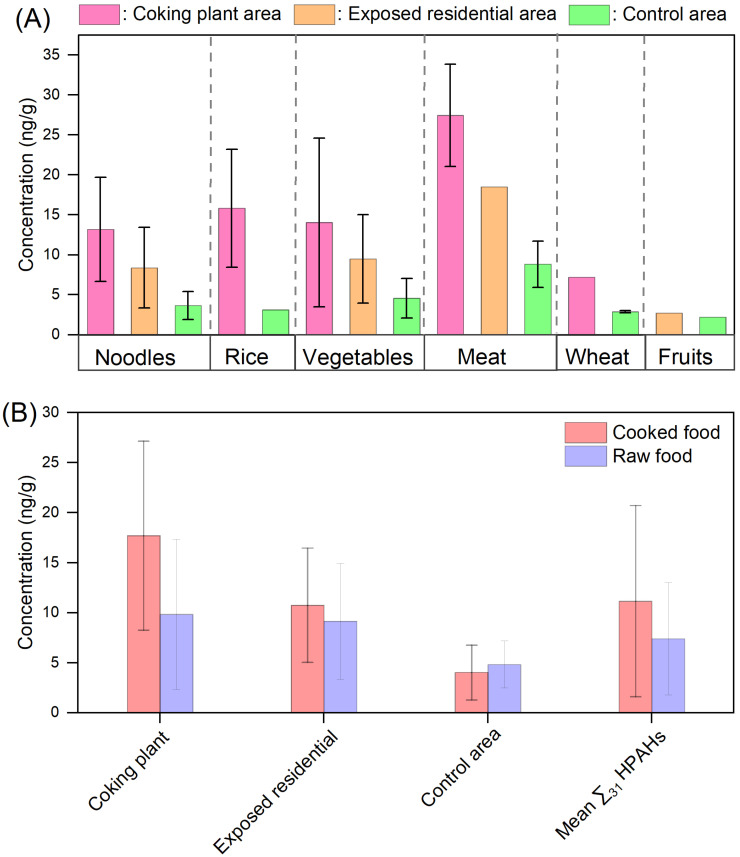
Concentrations of HPAHs in different dietary categories (**A**) and in cooked versus raw foods (**B**) among populations from the coking plant area, exposed residential area, and control area. Error bars indicate the variability among different raw and cooked food samples included in the analysis.

HPAH concentrations varied across food types and between cooked and raw foods (Figure 3). Meat had the highest levels, followed by vegetables, noodles, and rice. HPAHs such as 9-Cl-Fle, 5-Br-Ana, and 2-Br-Fle were consistently detected, whereas eight HPAHs were exclusive to coking plant area samples (1.35–4.02 ng/g). Cooked foods in the coking plant area exhibited approximately twofold higher HPAH concentrations than raw foods, whereas cooked and raw foods showed comparable concentration ranges in the exposed residential and control areas.

### 3.5. Dietary Cancer Risk of HPAHs

**Figure 4:**
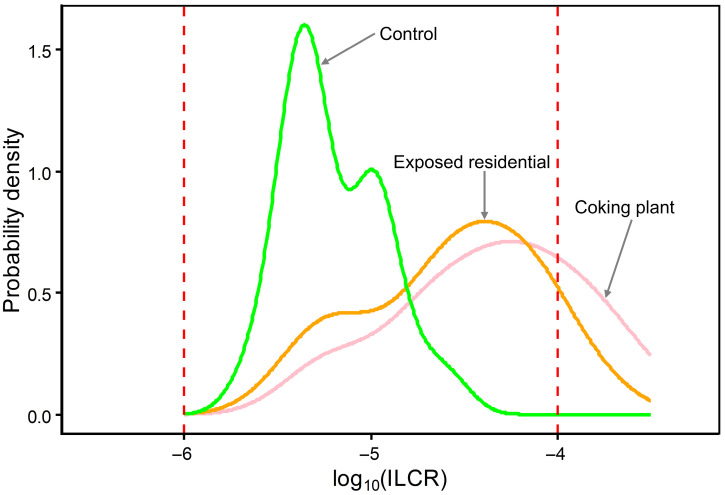
Probability density distributions of log_10_-transformed ILCR associated with dietary exposure to HPAHs in the coking plant area, exposed residential area, and control area. Kernel density estimation was applied to characterize population-level risk distributions without assuming a parametric form. Vertical dashed red lines denote the benchmark cancer risk thresholds of 10^−6^ (negligible risk) and 10^−4^ (unacceptable risk), respectively.

The probability density distributions of log10-transformed ILCR associated with dietary exposure to HPAHs exhibited pronounced spatial heterogeneity among the three study areas (Figure 4). The control area showed a narrowly clustered distribution centered around the 10^−6^ risk level, with the majority of the population remaining within the negligible cancer risk range. In contrast, both the coking plant area and the exposed residential area displayed right-shifted and broadened ILCR distributions, indicating elevated and more heterogeneous dietary cancer risks.

The coking plant area exhibited the most pronounced rightward shift, with a substantial fraction of the probability density extending toward and beyond the unacceptable risk threshold of 10^−4^. This long right tail suggests the presence of a subgroup with potentially elevated chronic dietary risk associated with higher toxicity-weighted HPAH exposure. The exposed residential area showed an intermediate risk profile, characterized by a broader distribution than the control area and a noticeable right tail approaching the unacceptable risk benchmark, albeit with a lower probability density than that observed in the coking plant area.

Notably, partial overlap in the probability density distributions was observed between the coking plant area and the exposed residential area, whereas minimal overlap occurred between either exposed group and the control area. This separation highlights the strong influence of proximity to industrial emission sources on dietary cancer risk from HPAHs. Formal statistical comparison of log_10_-transformed ILCR confirmed a significant overall difference among the three study areas (Kruskal–Wallis test, χ^2^ = 44.15, *p* = 2.59 × 10^−10^). Pairwise Wilcoxon rank-sum tests with Holm adjustment showed that both the coking plant area and the exposed residential area differed significantly from the control area (adjusted *p* < 0.001), whereas the difference between the coking plant area and the exposed residential area was not statistically significant (adjusted *p* > 0.05). Overall, the screening-level risk ranking inferred from the ILCR distributions followed the order: coking plant area > exposed residential area > control area, consistent with stronger industrial influence on toxicity-weighted dietary cancer-risk profiles.

### 3.6. In Silico Predicted Organ-Specific Toxicity of HPAHs

**Figure 5:**
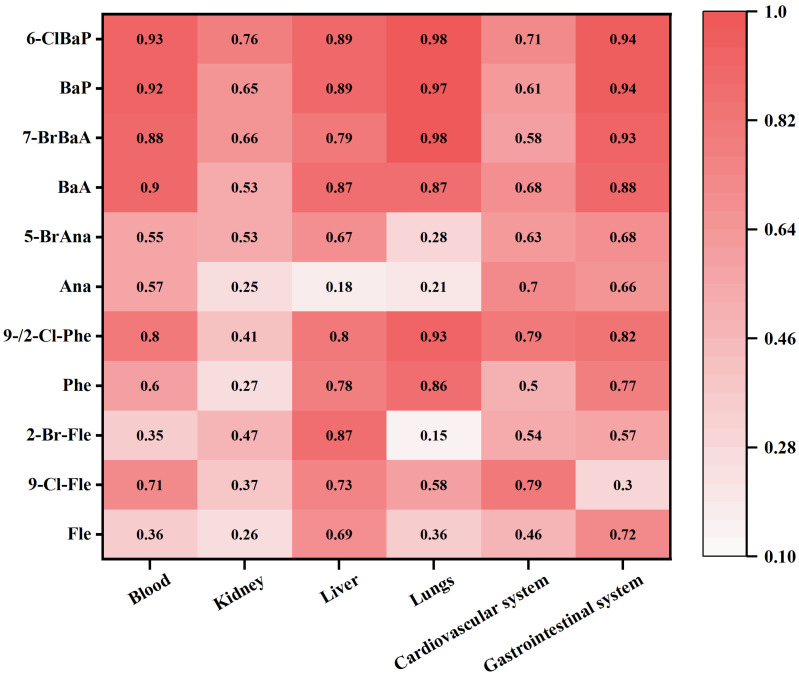
Predicted toxicological profiles of characteristic HPAHs based on ACD/Percepta modeling.

In silico toxicity predictions suggested that some low-detection HPAHs, such as 7-Br-BaA (1.24 ng/g) and 6-Cl-BaP (2.06 ng/g), may have relatively high organ-specific toxicological relevance (Figure 5). High-detection HPAHs, including 9-Cl-Fle, 5-Br-Ana, 2-Br-Fle, and 9-/2-Cl-Phe, also showed elevated predicted toxicity scores across multiple systems, including blood, cardiovascular, gastrointestinal, kidney, liver, and lung endpoints, except for 2-Br-Fle in liver (0.87) and 9-/2-Cl-Phe in kidney (0.4). These results should be interpreted as model-based toxicity indications that help prioritize potentially hazardous congeners, rather than direct evidence of observed biological effects under real-world dietary exposure conditions.

### 3.7. Sensitivity of ILCR Estimates to Exposure Assumptions

Sensitivity analysis showed that the absolute ILCR values changed predictably under alternative exposure scenarios. Compared with the base-case scenario, the lower-bound exposure scenario reduced ILCR values to 44.4% of the original estimates, whereas the upper-bound scenario increased ILCR values to 2.0-fold of the base-case values. Despite these changes in absolute magnitude, the overall between-group risk pattern remained unchanged, with the coking plant area consistently showing the highest toxicity-weighted dietary cancer-risk profile, followed by the exposed residential area and the control area (Table S6; Figure S3). These results indicate that the relative spatial contrast in dietary cancer risk is robust to plausible variation in fixed exposure assumptions.

## 4. Discussion

### 4.1. Spatial Patterns and Sources of HPAHs

The dietary HPAH profile exhibited a clear spatial gradient, with mean concentrations of 62.51 ng/g in the coking plant area, 33.68 ng/g in the exposed residential area, and 16.52 ng/g in the control area (Table S5). This pattern is consistent with stronger industrial influence on local food contamination in the vicinity of the coking plant. Direct comparison of the ∑HPAH concentrations observed in this study with those reported for other industrial regions worldwide remains difficult because methodologically comparable dietary datasets are extremely scarce. In particular, HPAH studies based on the duplicate diet method are, to our knowledge, very limited, whereas most published investigations from industrial areas have focused on environmental matrices such as air, soil, or sediment rather than prepared food samples. Accordingly, the ∑HPAH levels reported here should be interpreted primarily within the context of industrially influenced dietary exposure in the study area, while broader cross-regional benchmarking remains constrained by current data availability. Notably, the coking plant area contained 25 HPAHs, whereas 18 and 10 HPAHs were detected in the exposed residential and control areas, respectively, indicating a reduction in species diversity with distance from the emission source. The co-occurrence of eight HPAHs characteristic of the coking plant area and seven HPAHs found across all areas is consistent with multiple potential contamination pathways, including local industrial deposition and broader atmospheric transport to adjacent residential zones [31,32], although these pathways were not directly resolved in the present study. Mechanistically, halogenated PAHs are stabilized by electron-withdrawing halogens, reducing microbial degradation and promoting accumulation in both environmental media and food matrices. Detection of seven HPAHs not previously reported in food samples (e.g., 1,5-Cl2-Ant, 5-Br-Ana) underscores gaps in current monitoring and suggests that conventional PAH-focused assessments may underestimate dietary exposure. Because direct source-tracing analyses were not included, the observed spatial patterns should not be attributed exclusively to coking emissions, and contributions from other industrial activities, local environmental background, and food-related inputs cannot be fully excluded.

### 4.2. Influence of Food Matrices and Cooking on HPAH Accumulation

HPAH accumulation is strongly influenced by food composition and culinary practices. Lipid-rich foods, particularly meats, consistently exhibited the highest concentrations (e.g., 9-/2-Cl-Phe accounted for 13.7% of total HPAHs in cafeteria meals), followed by vegetables, noodles, and rice (Figure 3). Thermal processing amplified HPAH levels: cooked foods in the coking plant area displayed approximately twofold higher concentrations than raw foods (1.35–4.02 ng/g), whereas only minor differences were observed between cooked and raw foods in the exposed residential and control areas. This amplification may reflect the combined influence of cooking-related formation and stronger pre-existing contamination of raw ingredients in the coking plant area. In other words, high-temperature processing may enhance HPAH burdens more noticeably under conditions of greater industrial influence, whereas the weaker differences observed in the exposed residential and control areas suggest that de novo formation alone is unlikely to explain the full pattern [33,34]. Although meat samples showed higher HPAH concentrations than lower-fat food categories in the present study, fat content was not quantified for individual samples. Therefore, the current results should be interpreted as category-based exposure differences rather than direct evidence of a quantitative relationship between lipid content and HPAH levels. These findings indicate a synergistic effect whereby environmental contamination interacts with cooking practices to increase dietary exposure, indicating the importance of considering food processing in exposure assessments.

### 4.3. Molecular Weight, Halogenation, and Toxicity Profiles

HPAHs exhibit ring-number-dependent behavior and toxicity. Low-molecular-weight (LMW, 3-ring) HPAHs were most abundant due to higher volatility and solubility, whereas medium- (MMW, 4-ring) and high-molecular-weight (HMW, 5-ring) species, though less frequent, displayed higher toxic equivalency factors (TEFs) and greater carcinogenic potential (Figure S2). Halogen substitution is generally associated with increased lipophilicity, chemical stability, and binding affinity to biomolecules, which may contribute to greater toxicological concern [35,36]. ACD/Percepta predictions further suggested elevated organ-specific toxicity potential even for low-abundance compounds such as 7-Br-BaA (1.24 ng/g) and 6-Cl-BaP (2.06 ng/g), particularly for blood, liver, kidney, and cardiovascular endpoints. However, these results are based on in silico modeling and should be interpreted as toxicity indications for congener prioritization rather than direct evidence of observed organ damage. To provide additional context, we compared the compositional patterns of 15 parent PAHs previously measured in canteen food samples from the same coking-plant setting with the HPAH patterns observed in the present study. The comparison showed that fluoranthene and phenanthrene contributed 4.6% and 4.3% of the total parent-PAH concentration, respectively, whereas halogenated derivatives of fluorene, phenanthrene, and anthracene accounted for a much larger proportion of total HPAH concentrations in canteen foods. This contrast suggests that parent PAHs and HPAHs may display different compositional behaviors under industrially influenced food-preparation conditions. However, because fully matched parent-PAH data were not available for the same batch of samples, these observations should be interpreted as comparative compositional patterns rather than evidence of direct correlation or transformation relationships. These results suggest that assessments based solely on concentration or detection frequency may substantially underestimate health risks associated with halogenated HPAHs.

### 4.4. Dietary Cancer Risk and Cumulative Exposure

The elevated dietary cancer risks observed in populations residing near the coking plant are consistent with the combined influence of source proximity and toxicity-weighted cumulative exposure to halogenated PAHs. While mean ILCR values indicate substantially higher risk levels in the coking plant and exposed residential areas compared with the control area, the pronounced right-skewed distributions of ILCR (Figure 4) further suggest the presence of a subgroup with potentially elevated dietary cancer risk. This distributional feature underscores that population averages alone may underestimate the health burden borne by susceptible individuals.

Importantly, cumulative dietary cancer risk was not driven solely by the most abundant HPAHs, but was strongly influenced by congeners with high carcinogenic potency. As reflected by the congener-specific contributions to TEQ-adjusted risk (Figure 2), several low-detection yet highly toxic compounds dominated cumulative ILCR, demonstrating that concentration-based assessments alone are insufficient for evaluating dietary hazards in complex industrial emission scenarios.

The dominance of specific halogenated fluoranthene, phenanthrene, and anthracene derivatives in cumulative risk is consistent with industrially influenced contamination profiles in the study area, although direct source-tracing evidence was not available to attribute these patterns specifically to coking emissions. Dietary intake of locally sourced foods may integrate multiple pathways, including atmospheric deposition and food contamination, into a cumulative exposure burden, consistent with previous observations in industrially impacted regions.

Overall, these results highlight that dietary cancer risk in industrial settings is likely influenced by the interaction of emission source characteristics, food contamination pathways, and congener-specific toxicity. Risk management strategies should therefore prioritize not only reductions in total HPAH concentrations but also targeted control of highly potent congeners that dominate cumulative cancer risk.

### 4.5. Emerging HPAHs and Database Implications

The identification of seven previously unreported HPAHs expands the coverage of dietary exposure databases and highlights limitations in existing regulatory frameworks, which often omit halogenated derivatives (Section 3.1). Many newly detected compounds lack TEFs, complicating conventional cumulative risk estimation. By incorporating these species into toxicity-weighted models, hazard estimation accuracy is improved, and emerging contaminants are better represented in exposure assessments. This study thus provides mechanistic insight into halogenated PAHs in industrially influenced diets and establishes a foundation for future monitoring and regulatory updates.

### 4.6. Mechanistic Insights and Risk Management

**Figure 6:**
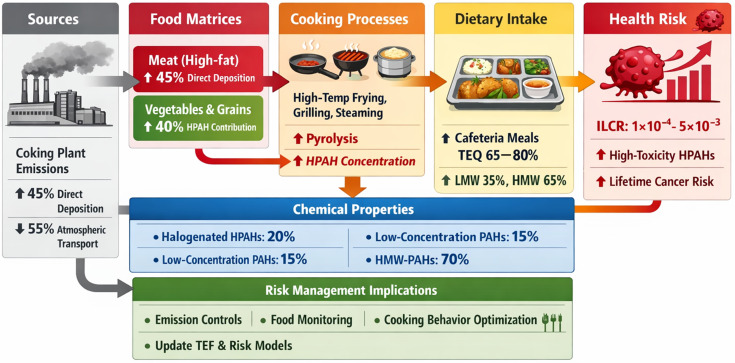
Mechanistic pathway of HPAH contamination from coking plant emissions to dietary exposure and associated cancer risk. Emissions deposit on high-fat foods and are transported atmospherically to vegetables and grains. Cooking processes (frying, grilling, steaming) increase HPAH concentrations. Chemical properties, including halogenation and molecular weight, modulate persistence and toxicity. Dietary intake, especially from cafeteria meals, contributes substantially to TEQ and lifetime cancer risk. Risk management includes emission control, food monitoring, and cooking behavior optimization.

Dietary accumulation of HPAHs results from the interplay of industrial emissions, chemical persistence, and food processing (Figure 6). Contamination in the coking plant area may be influenced by local industrial emissions through pathways such as particulate deposition onto raw ingredients, although these pathways were not directly traced in the present study. Coking plant emissions contribute directly via particulate deposition and indirectly by enhancing adherence to raw ingredients. High-temperature cooking, especially of lipid-rich foods in canteens, may further increase halogenated HPAHs (Figure 6). Halogen substitution enhances persistence and organ-specific toxicity, as confirmed by ACD/Percepta predictions (Figure 6). Conventional assessments based solely on PAH concentrations likely underestimate risk, particularly for low-abundance but highly toxic species (Figure 6).

The detection of previously unreported HPAHs emphasizes the need to refine cumulative risk models and update TEF databases. Effective mitigation should integrate emission reduction, monitoring of persistent halogenated PAHs in food matrices, and consideration of cooking practices in risk communication. Because the highest-exposure group represented a real-world coking plant setting that may involve both occupational and residential influence, these two factors could not be fully disentangled in the present sampling design. Accordingly, the observed between-group differences should be interpreted as exposure contrasts across industrial influence scenarios, rather than effects attributable exclusively to either occupation or residence. In addition, each participant provided only a one-day duplicate diet sample. Although the duplicate diet method is widely regarded as a robust approach for measuring actual dietary intake on the sampling day, it does not capture day-to-day or seasonal variability in food choices, cooking practices, or contaminant concentrations. Therefore, the exposure and ILCR estimates reported here should be interpreted as screening-level indicators of chronic dietary risk under real-world industrial exposure scenarios, rather than precise long-term risk estimates for specific individuals. Future studies incorporating repeated sampling across multiple days and seasons would help better constrain temporal variability and strengthen chronic exposure characterization. Additional uncertainty arises from the use of QSAR-derived REP_BaP values for congeners lacking experimental toxicological data, which may affect the absolute magnitude of TEQ and ILCR estimates. In particular, the CTV model used for QSAR-assisted extrapolation has an average prediction error of approximately 0.97 log_10_ units, indicating that uncertainty in REP_BaP estimates may span roughly one order of magnitude for some congeners. While Figure 4 reflects the variability of ILCR distributions across dietary samples under the adopted risk-assessment framework, uncertainty in QSAR-derived REP_BaP values represents a separate source of model-input uncertainty that may influence the absolute magnitude of TEQ and ILCR estimates. Additional uncertainty also arises from the treatment of concentrations below the limit of detection. In the present study, non-detects were treated as zero in the primary analysis as a lower-bound assumption, providing a conservative basis for estimating concentration, TEQ, and ILCR but potentially underestimating their absolute magnitude for congeners with low detection frequencies. Uncertainty also stems from the use of fixed exposure assumptions in the ILCR model, including dietary ingestion rate, body weight, and exposure duration. However, the scenario-based sensitivity analysis showed that plausible variation in these exposure parameters changed the absolute magnitude of ILCR but did not alter the relative spatial pattern of toxicity-weighted dietary risk across the three study areas. In addition, the organ-specific toxicity patterns inferred from ACD/Percepta should be interpreted as in silico toxicity indications for congener prioritization, rather than definitive evidence of organ damage under real-world dietary exposure conditions. Accordingly, the present study distinguishes among directly measured contamination patterns in duplicate diet samples, model-based estimates of toxicity-weighted cancer risk and organ-specific toxicity potential, and broader inferences regarding industrial influence, food contamination pathways, and health implications. Collectively, these findings indicate that protecting vulnerable populations requires a holistic understanding of contaminant sources, transformation processes, and dietary behavior, rather than reliance on single-factor assessments, providing a quantitative and mechanistic foundation for long-term carcinogenic and organ-specific risk management (Figure 6).

## 5. Conclusions

This study presents a comprehensive assessment of dietary exposure to 31 halogenated polycyclic aromatic hydrocarbons (HPAHs), including seven previously unreported congeners, in populations residing near a coking industrial area. Lipid-rich foods showed the highest contamination levels, while cooking-related amplification of dietary HPAH burdens was most evident in the coking plant area. Although several HPAHs occurred at low concentrations, congeners with high toxic equivalency factors contributed disproportionately to cumulative dietary cancer risk. In silico toxicity predictions further indicated potential associations with adverse effects in the blood, liver, kidney, and cardiovascular systems. Collectively, these findings suggest that industrial influence, food composition, and culinary practices jointly contribute to dietary exposure patterns and toxicity-weighted cancer risk. Incorporating halogenated PAHs into routine monitoring programs and applying toxicity-weighted risk assessment frameworks may improve the accuracy of dietary risk assessments in industrially influenced regions.

## Data Availability

The original contributions presented in this study are included in the article/Supplementary Materials. Further inquiries can be directed to the corresponding author.

## Supplementary Materials

The following supporting information can be downloaded at https://www.mdpi.com/article/10.3390/jox16030079/s1: Figure S1: Schematic map of the study area in Lüliang City, Shanxi Province, northern China, showing the relative locations of the coking plant area, exposed residential area, and control area. The approximate distances between the coking plant area and the exposed residential area and control area are ~1 km and ~50 km, respectively. To protect sensitive information related to industrial infrastructure, exact sampling locations and coordinates are not shown, and the map is provided for illustrative purposes only; Figure S2: Concentrations of HPAHs in the diets of populations in coking plants, exposed residential areas and control areas at three different molecular weights; Figure S3: Sensitivity of median ILCR values to alternative exposure assumptions across the coking plant area, exposed residential area, and control area. Lower- and upper-bound scenarios were generated by varying dietary ingestion rate, body weight, and exposure duration relative to the base-case assumptions; Table S1: Questionnaire used in the duplicate diet method to ensure representative sampling and to capture habitual dietary patterns and regional cooking practices; Table S2: Food samples collected across different areas in Shanxi Province, China, with corresponding food types, for representative dietary exposure assessment using the duplicate diet method; Table S3: Validation results for the determination of halogenated polycyclic aromatic hydrocarbons (HPAHs) in dietary samples, including method recoveries, relative standard deviations (RSD), limits of detection (LOD), and limits of quantification (LOQ); Table S4: The reference toxic equivalent factors (REPBaP) of HPAHs comprised in carcinogenic risk assessment; Table S5: Concentrations of 31 HPAHs in the diets of different populations in this study of Shanxi Province, China (ng/g); Table S6: Sensitivity analysis of ILCR estimates under alternative exposure scenarios for populations in the coking plant area, exposed residential area, and control area.
