# Supplementary material for: Congener-Specific Dietary Exposure and Predicted Organ-Specific Toxicity of Halogenated PAHs in Populations Living near a Coking Industrial Area
Source: J Xenobiot. 2026 May 5;16(3):79. doi: 10.3390/jox16030079 (PMC13214609; doi:10.3390/jox16030079)
Supplement: Supplementary file 1 [file jox-16-00079-s001.zip › jox-4232593-supplementary.pdf]

Supplementary material

Congener-Specific Dietary Exposure and Predicted Organ-Specific Toxicity of Halogenated PAHs in Populations Living near a Coking Industrial Area

Yanpeng Gao [1,2], Weijie Lu [1,2], Yibo Zhang [1,2], Mingze Geng [1,2], Xianglong Luo [1,2], Yuemeng Ji [1,2] and Yang-Guang Gu [3,*]

1 Guangdong-Hong Kong-Macao Joint Laboratory for Contaminants Exposure and Health, Guangdong Key Laboratory of Environmental Catalysis and Health Risk Control, Institute of Environmental Health and Pollution Control, Guangdong University of Technology, Guangzhou 510006, China; gaoyp2016@gdut.edu.cn (Y.G.); 13650615863@163.com (W.L.); 3123005518@mail2.gdut.edu.cn (Y.Z.); 13753833198@163.com (M.G.); luoxl0211@163.com (X.L.); jiym@gdut.edu.cn (Y.J.)

2 Guangdong Basic Research Center of Excellence for Ecological Security and Green Development, Key Laboratory of City Cluster Environmental Safety and Green Development of the Ministry of Education, School of Environmental Science and Engineering, Guangdong University of Technology,

Guangzhou 510006, China

3 South China Sea Fisheries Research Institute, Chinese Academy of Fishery Sciences,

Guangzhou 510300, China

* Correspondence: hydrobio@163.com; Tel.: +86-20-89102080

Email address: sunshinegu@scsfri.ac.cn; hydrobio@163.com (Gu)

Contents

1. Supplementary figures 4

Figure S1. Schematic map of the study area in Lüliang City, Shanxi Province, northern China, showing the relative locations of the coking plant area, exposed residential area, and control area. The approximate distances between the coking plant area and the exposed residential area and control area are ~1 km and ~50 km, respectively. To protect sensitive information related to industrial infrastructure, exact sampling locations and coordinates are not shown, and the map is provided for illustrative purposes only. 4

Figure S2. Concentrations of HPAHs in the diets of populations in coking plants, exposed residential areas and control areas at three different molecular weights. 5

Figure S3. Sensitivity of median ILCR values to alternative exposure assumptions across the coking plant area, exposed residential area, and control area. Lower- and upper-bound scenarios were generated by varying dietary ingestion rate, body weight, and exposure duration relative to the base-case assumptions. 6

2. Supplementary tables 7

Table S1. Questionnaire used in the duplicate diet method to ensure representative sampling and to capture habitual dietary patterns and regional cooking practices. ... 7

Table S2. Food samples collected across different areas in Shanxi Province, China, with corresponding food types, for representative dietary exposure assessment using the duplicate diet method. 9

Table S3. Validation results for the determination of halogenated polycyclic aromatic hydrocarbons (HPAHs) in dietary samples, including method recoveries, relative standard deviations (RSD), limits of detection (LOD), and limits of quantification (LOQ). 13

Table S4. The reference toxic equivalent factors (REP_{BaP}) of HPAHs comprised in carcinogenic risk assessment. 14

Table S5. Concentrations of 31 HPAHs in the diets of different populations in this study of Shanxi Province, China(ng/g) 15

Table S6. Sensitivity analysis of ILCR estimates under alternative exposure scenarios for populations in the coking plant area, exposed residential area, and control area. 17

1. Supplementary figures

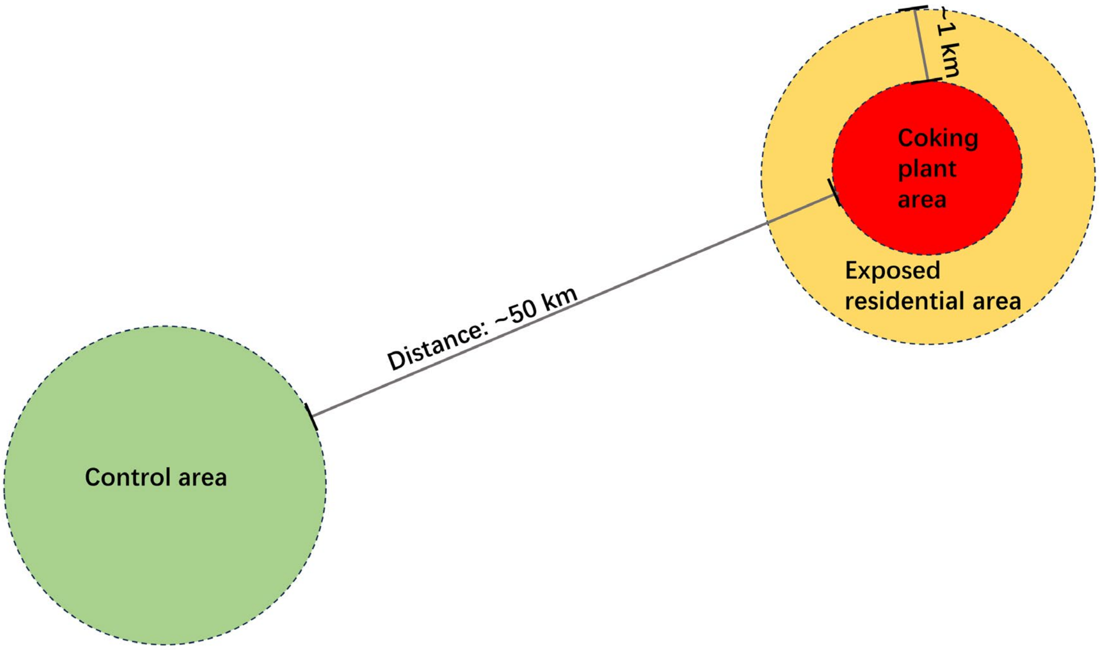

Figure S1. Schematic map of the study area in Lüliang City, Shanxi Province, northern China, showing the relative locations of the coking plant area, exposed residential area, and control area. The approximate distances between the coking plant area and the exposed residential area and control area are ~1 km and ~50 km, respectively. To protect sensitive information related to industrial infrastructure, exact sampling locations and coordinates are not shown. The figure is provided for illustrative purposes only and is not intended for quantitative spatial interpretation.

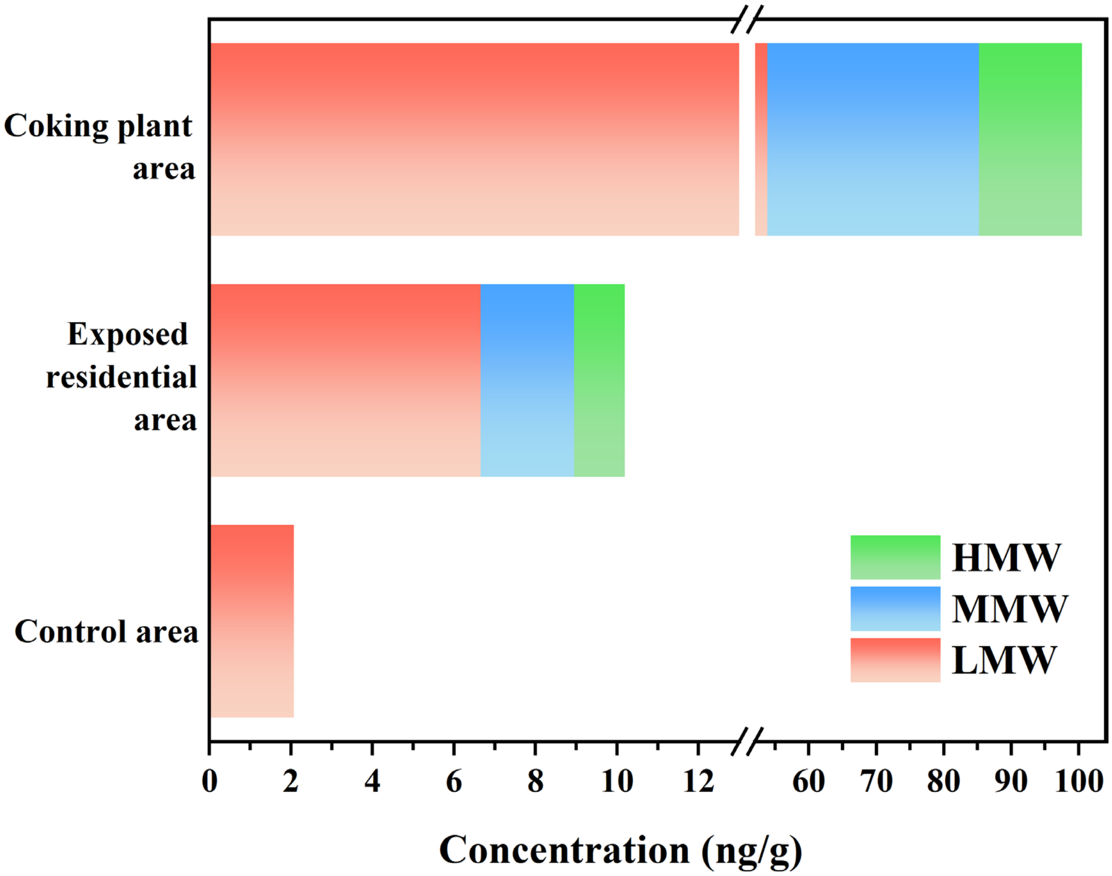

Figure S2. Concentrations of HPAHs in the diets of populations in coking plants, exposed residential areas and control areas at three different molecular weights.

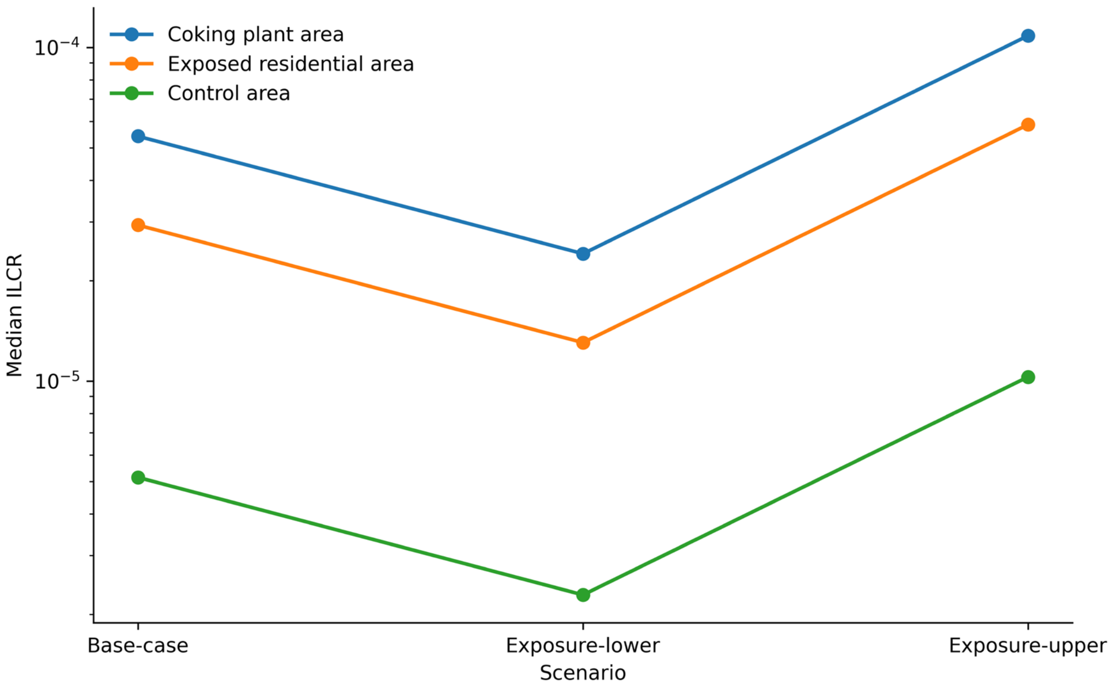

Figure S3. Sensitivity of median ILCR values to alternative exposure assumptions across the coking plant area, exposed residential area, and control area. Lower- and upper-bound scenarios were generated by varying dietary ingestion rate, body weight, and exposure duration relative to the base-case assumptions.

2. Supplementary tables

Table S1. Questionnaire used in the duplicate diet method to ensure representative sampling and to capture habitual dietary patterns and regional cooking practices.

**A01 Number: ______ Gender: _____ Age: ______ Weight: ______kg**
**A02 Collection time: __________ A03 Place of collection: _____________**
**A04 Living habit and family history:**
**Smoke:** ☐ Yes ☐ No ☐ Has quit smoking for_____years<br>**Drink wine:** ☐ Yes ☐ No ☐ Has quit drinking for_____years<br>**Family history:** ☐ No ☐ Father ☐ Mother ☐ Other person__________
**A05 Kitchen Information:**
**Whether to cook yourself:** ☐ Yes ☐ No<br>**Do you have a kitchen where you live:** ☐ Yes, with the living room serialization<br>☐ Yes, separate kitchen ☐ No<br>**What type of fuel do you mainly use in the kitchen where you live? (Multiple choice):**<br>☐ Coal ☐ firewood ☐ Liquefied Petroleum Gas ☐ Natural Gas ☐ Biogas ☐ Electricity<br>☐ Other________<br>**What is the main exhaust device used in the kitchen where you live:**<br>☐ Chimneys ☐ Exhaust fans ☐ Range Hoods ☐ Neither<br>**How long do you usually spend in the kitchen for cooking:** ________min
**A06 Sample information and raw material sources:**
**Breakfast:** __________________________ Cooking method: ________<br>Weight: ______kg<br>（☐ Local ____________ ☐ Field___________）<br>**Lunch:** _____________________________ Cooking method: ________<br>Weight: ______kg<br>☐ Staple food（☐ Local ____________ ☐ Field___________）<br>☐ Vegetables（☐ Local ____________ ☐ Field___________）<br>☐ Meat（☐ Local ____________ ☐ Field___________）<br>**Snacks:** _____________________________ Weight: ______kg<br>**Dinner:** _____________________________ Cooking method: ________<br>Weight: ______kg<br>（☐ Local ____________ ☐ Field___________）
**A07 Local Residence History:**
☐2-3 years ☐4-5 years ☐6-10 years ☐>10 years<br>☐Within 1 year (relocated from another location)
**A08 Cooking habits (common cooking style):**
☐ Frying ☐ deep fry ☐ stir-fry ☐ Roast ☐ Steaming ☐ Cook ☐ marinade<br>☐ brine ☐ Smoke ☐ braised ☐ mix
**A09 Have the habit of eating late night snacks:**
☐ Yes,________times a week ☐ No
**A10 Eating habits:**

Food	Never	1 time/week	2-3 times/week	4-6 times/week	Everyday
Rice	☐	☐	☐	☐	☐
Noodles	☐	☐	☐	☐	☐
Beans and their products	☐	☐	☐	☐	☐
Vegetables and vegetable products	☐	☐	☐	☐	☐
Meat and meat products	☐	☐	☐	☐	☐
Aquatic products and their products	☐	☐	☐	☐	☐
Dairy, eggs and their products	☐	☐	☐	☐	☐
Tea drinking	☐	☐	☐	☐	☐
Fried food and barbecue	☐	☐	☐	☐	☐
Overnight food	☐	☐	☐	☐	☐

Table S2. Food samples collected across different areas in Shanxi Province, China, with corresponding food types, for representative dietary exposure assessment using the duplicate diet method.

Serial number	Sampling area	Food type
1	Coking plant area(Village)	Noodles
2	Coking plant area(Village)	Rice
3	Coking plant area(Village)	Noodles
4	Coking plant area(Village)	Noodles
5	Coking plant area(Village)	Noodles
6	Coking plant area(Village)	Soup
7	Coking plant area(Village)	Noodles
8	Coking plant area(Village)	Restaurant food
9	Coking plant area(Village)	Restaurant food
10	Coking plant area(Village)	Restaurant food
11	Coking plant area(Village)	Bread
12	Coking plant area(Village)	Noodles
13	Coking plant area(Village)	Tofu and potato
14	Coking plant area(Village)	Green vegetables
15	Coking plant area(Village)	Garlic
16	Coking plant area(Village)	Green vegetables
17	Coking plant area(Village)	Pepper
18	Coking plant area(Village)	Leaf mustard
19	Coking plant area(Village)	Walnut
20	Coking plant area(Village)	Green vegetables
21	Coking plant area(Village)	Pepper
22	Coking plant area(Village)	Leaf mustard
23	Coking plant area(Canteen)	Noodles
24	Coking plant area(Canteen)	Stewed vegetables
25	Coking plant area(Canteen)	Meatball
26	Coking plant area(Canteen)	Noodles

27	Coking plant area(Canteen)	Noodles
28	Coking plant area(Canteen)	Noodles
29	Coking plant area(Canteen)	Green vegetables
30	Coking plant area(Canteen)	Rice
31	Coking plant area(Canteen)	Rice
32	Control area(Worker)	Noodles
33	Control area(Worker)	Noodles
34	Control area(Worker)	Dumplings
35	Control area(Worker)	Noodles
36	Control area(Worker)	Noodles
37	Control area(Worker)	Tofu and agaric
38	Control area(Worker)	Milk
39	Control area(Worker)	Noodles
40	Control area(Worker)	Noodles
41	Exposed residential area(Village）	Steamed oats noodle
42	Exposed residential area(Village）	Dried bean curd
43	Exposed residential area(Village）	Pork
44	Exposed residential area(Village）	Noodles
45	Exposed residential area(Village）	celery
46	Exposed residential area(Village）	Apple
47	Exposed residential area(Village）	Bean sprout
48	Exposed residential area(Village）	Baby cabbage
49	Exposed residential area(Village）	Zucchini
50	Exposed residential area(Village）	Romaine lettuce
51	Exposed residential area(Village）	Cabbage
52	Exposed residential area(Village）	Green pepper
53	Exposed residential area(Village）	Pepper
54	Exposed residential area(Village）	Lettuce
55	Exposed residential area(Village）	Romaine lettuce

56	Exposed residential area(Village）	Cabbage
57	Exposed residential area(Village）	Green vegetables
58	Exposed residential area(Village）	Tomato
59	Control area(Taiyuan City)	Noodles
60	Control area(Taiyuan City)	Dumplings
61	Control area(Taiyuan City)	Noodles
62	Control area(Qingxu County)	Roast duck
63	Control area(Qingxu County)	Deep-fried meat
64	Control area(Qingxu County)	Dumplings
65	Control area(Qingxu County)	Green vegetables
66	Control area(Ordinary resident)	Romaine lettuce
67	Control area(Ordinary resident)	cauliflower
68	Control area(Ordinary resident)	Lettuce
69	Control area(Ordinary resident)	Baby cabbage
70	Control area(Ordinary resident)	Coriander
71	Control area(Ordinary resident)	Leek
72	Control area(Ordinary resident)	Broccoli
73	Control area(Ordinary resident)	Noodles
74	Control area(Ordinary resident)	Noodles
75	Control area(Ordinary resident)	Pork
76	Control area(Ordinary resident)	Romaine lettuce
77	Control area(Ordinary resident)	Broccoli
78	Control area(Ordinary resident)	Kidney bean
79	Control area(Ordinary resident)	Potato
80	Control area(Ordinary resident)	Carrot
81	Control area(Ordinary resident)	Potato
82	Control area(Ordinary resident)	Garlic
83	Control area(Ordinary resident)	Green pepper
84	Control area(Ordinary resident)	Noodles

85	Control area(Ordinary resident)	Pepper
86	Control area(Ordinary resident)	Zucchini
87	Control area(Ordinary resident)	Rice

Table S3. Validation results for the determination of all target halogenated polycyclic aromatic hydrocarbons (HPAHs) analyzed in dietary samples, including the seven previously unreported congeners, showing method recoveries, relative standard deviations (RSD), limits of detection (LOD), and limits of quantification (LOQ).

HPAHs	Recovery (%)	RSD (%)	LOD (ng/g)	LOQ (ng/g)
9-Cl-Fle	93.33	7.85	0.0068	0.0187
5-Br-Ana	85.35	8.56	0.0010	0.0055
2-Br-Fle	89.01	10.27	0.0010	0.0054
9-/2-Cl-Phe	102.03	9.33	0.0478	0.1222
1-/2-Cl-Ant	106.90	6.19	0.0431	0.1075
9-Cl-Ant	105.79	6.08	0.0432	0.1076
1,2-Br_2-Any	92.22	6.34	0.0052	0.0156
2,7-Cl_2-Fle	81.79	3.21	0.0073	0.0416
3-Br-Phe	79.24	3.81	0.0075	0.0425
9-Br-Phe	90.38	10.08	0.0057	0.0143
2-Br-Phe	98.02	8.24	0.0048	0.0111
1-Br-Ant	96.90	9.37	0.0109	0.0388
9-Br-Ant	79.82	9.35	0.0117	0.0391
1,5-Cl_2-Ant	102.67	4.63	0.0050	0.0178
9,10-Cl_2-Ant	101.35	6.28	0.0063	0.0169
9,10-Cl_2-Phe	95.01	9.44	0.0073	0.0162
2,7-Br_2-Fle	84.28	7.37	0.0109	0.0385
3-Cl-Fluo	80.16	7.85	0.0058	0.0190
1-Cl-Pyr	85.73	5.60	0.0050	0.0164
3-Br-Fluo	84.43	10.68	0.0046	0.0200
9,10-Br_2-Ant	98.49	7.63	0.0131	0.0440
4-Br-Pyr	94.53	5.63	0.0017	0.0071
1-Br-Pyr	102.01	8.62	0.0031	0.0134
9,10-Br_2-Phe	107.46	10.04	0.0133	0.0420
7-Cl-BaA	81.06	6.90	0.0032	0.0154
1,5,9,10-Cl_4-Ant	80.04	6.87	0.0099	0.0401
1,6-Br_2-Pyr	80.93	0.68	0.0038	0.0152
7-Br-BaA	79.47	1.06	0.0038	0.0135
6-Cl-BaP	93.68	10.94	0.0073	0.0188

Table S4. The reference toxic equivalent factors (REP_{BaP}) of HPAHs comprised in carcinogenic risk assessment.

Compound Name	CAS	rings	REP_{BaP}	Reference compound
9-ClFle	6630-65-5	3	0.021	2-Br-Fle
5-BrAna	2051-98-1	3	0.022	2-Br-Fle
2-BrFle	1133-80-8	3	0.02	
9-ClPhe	947-72-8	3	0.03	
2-ClPhe	24423-11-8	3	0.03	
1-ClAnt[a]	4985-70-0	3	0.04	
2-ClAnt[a]	17135-78-3	3	0.10	
9-ClAnt	716-53-0	3	0.03	
2,7-Cl_2Fle	7012-16-0	3	0.022	2-Br-Fle
1,2-Br_2Any	14209-08-6	3	0.05	1,9-Cl_2-Phe
3-BrPhe	715-50-4	3	0.018	9-Br-Phe
9-BrPhe	573-17-1	3	0.02	
2-BrPhe	62162-97-4	3	0.02	
1-BrAnt	7397-92-4	3	0.05	
9-BrAnt	1564-64-3	3	0.01	
1,5-Cl_2Ant	6406-96-8	3	0.14	9,10-Cl_2-Ant
9,10-Cl_2Ant	605-48-1	3	0.2	
9,10-Cl_2Phe	17219-94-2	3	0.16	
2,7-Br_2Fle	16433-88-8	3	0.046	2-Br-Fle
3-ClFluo	25911-51-7	4	0.17	
1-ClPyr	34244-14-9	4	0.1	
3-BrFluo	13438-50-1	4	0.15	3-Cl-Fluo
9,10-Br_2Ant	523-27-3	3	0.18	9,10-Cl_2-Ant
4-BrPyr	1732-26-9	4	0.04	1-Br-Pyr
9,10-Br_2Phe	15810-15-8	4	0.15	9,10-Cl_2-Phe
1-BrPyr	1714-29-0	3	0.04	
7-ClBaA	20268-52-4	4	0.83	
1,5,9,10-Cl_4Ant	82843-47-8	3	0.3	9,10-Cl_2-Ant
1,6-Br_2Pyr	27973-29-1	4	0.045	1-Br-Pyr
7-BrBaA	32795-84-9	4	0.84	
6-ClBaP	21248-01-1	5	0.09	

Note: When calculating the TEQ of 1-ClAnt and 2-ClAnt, take the average of 0.4 and 1, which is 0.7; CTV predictions or calculation from CTV results: http://toxvalue.org.

Table S5. Concentrations of 31 HPAHs in the diets of different populations in this study of Shanxi Province, China(ng/g)

HPAHs	REP_{BaP}	Detection frequency(%)	Total samples N =87		Coking plant area N = 31		Exposed residential area N = 18		Control area N = 38	
			Mean	SD	Mean	SD	Mean	SD	Mean	SD
9-Chlorofluorene (9-Cl-Fle)	0.021	94.3%	1.67	1.40	2.24	1.85	1.56	0.87	1.20	0.82
5-Bromoacenaphthene (5-Br-Ana)	0.022	98.9%	1.84	1.55	1.97	1.86	1.89	1.11	1.71	1.43
2-Bromofluorene (2-Br-Fle)	0.02	77.0%	1.08	0.57	1.18	0.65	1.08	0.50	0.99	0.50
9-/2-Chlorophenanthrene (9-/2-Cl-Phe)	0.03	33.3%	6.79	2.36	8.24	2.29	5.15	0.52	4.69	0.26
1-/2-Chloroanthracene (1-/2-Cl-Ant)	0.07	3.4%	5.83	0.67	5.40	0.00	6.05	0.73		
9-Chloroanthracene (9-Cl-Ant)	0.03	2.3%	5.32	0.14	5.45	0.00	5.18	0.00		
1,2-DibroMoacenaphthylene (1,2-Br_2-Any)	0.05	5.7%	1.35	0.29	1.35	0.29				
2,7-Dichlorofluorene (2,7-Cl_2-Fle)	0.022	2.3%	2.09	0.55					2.09	0.55
3-Bromophenanthrene (3-Br-Phe)	0.018	3.4%	1.65	0.13			1.60	0.00	1.67	0.15
9-Bromophenanthrene (9-Br-Phe)	0.02	2.3%	2.13	0.29	2.13	0.29				
2-Bromophenanthrene (2-Br-Phe)	0.02	2.3%	4.02	2.80	4.02	2.80				
1-Bromoanthracene (1-Br-Ant)	0.05	1.1%	2.30	0.00	2.30	0.00				
9-Bromoanthracene (9-Br-Ant)	0.01	4.6%	2.99	0.87	3.86	0.05	2.13	0.01		
1,5-Dichloroanthracene (1,5-Cl_2-Ant)	0.14	12.6%	1.33	0.55	1.53	0.56	0.98	0.27		
9,10-Dichloroanthracene (9,10-Cl_2-Ant)	0.2	13.8%	1.76	0.83	1.89	0.85	1.49	0.70		
9,10-Dichlorophenanthrene (9,10-Cl_2-Phe)	0.16	13.8%	2.34	1.17	2.46	1.11	2.11	1.25		
2,7-Dibromofluorene (2,7-Br_2-Fle)	0.046	20.7%	2.98	1.58	3.23	1.78	2.16	0.15	2.92	0.00
3-Chlorofluoranthrene (3-Cl-Fluo)	0.17	5.7%	1.18	0.22	1.27	0.13	0.80	0.00		
1-Chloropyrene (1-Cl-Pyr)	0.1	0%								

3-Bromofluoranthene (3-Br-Fluo)	0.15	0%								
9,10-Dibromoanthracene (9,10-Br_2-Ant)	0.18	4.6%	3.48	0.97	3.48	0.97				
4-Bromopyrene (4-Br-Pyr)	0.04	14.9%	0.44	0.14	0.37	0.06	0.36	0.00	0.66	0.12
1-Bromopyrene (1-Br-Pyr)	0.04	9.2%	2.22	0.99	2.22	0.99				
9,10-Dibromophenanthrene (9,10-Br_2-Phe)	0.15	0%								
7-Chlorobenzo[a]anthracene (7-Cl-BaA)	0.83	0%								
1,5,9,10-Tetrachloranthene (1,5,9,10-Cl_4-Ant)	0.3	1.1%	3.07	0.00	3.07	0.00				
1,6-Dibromopyrene (1,6-Br_2-Pyr)	0.045	8.0%	0.60	0.09	0.64	0.11	0.51	0.00	0.58	0.0002
7-Bromobenzo[a]anthracene (7-Br-BaA)	0.84	5.7%	1.24	1.16	2.14	1.42	0.63	0.02		
6-Chlorobenzo[a]pyrene (6-Cl-BaP)	0.09	3.4%	2.06	0.30	2.06	0.30				
$\sum_{31}$HPAH			61.76		62.51		33.68		16.52	
TEQ			5.43		6.26		2.46		0.49	

Table S6. Sensitivity analysis of ILCR estimates under alternative exposure scenarios for populations in the coking plant area, exposed residential area, and control area.

Scenario	Area	N	Mean ILCR	Median ILCR	SD ILCR	Min ILCR	Max ILCR
Base-case	Coking plant area	3.10E+01	7.55E-05	5.42E-05	7.77E-05	4.92E-06	3.14E-04
Base-case	Exposed residential area	1.80E+01	4.30E-05	2.94E-05	4.00E-05	4.39E-06	1.62E-04
Base-case	Control area	3.80E+01	7.66E-06	5.15E-06	5.57E-06	2.07E-06	2.85E-05
Exposure-lower	Coking plant area	3.10E+01	3.36E-05	2.41E-05	3.45E-05	2.19E-06	1.40E-04
Exposure-lower	Exposed residential area	1.80E+01	1.91E-05	1.30E-05	1.78E-05	1.95E-06	7.20E-05
Exposure-lower	Control area	3.80E+01	3.41E-06	2.29E-06	2.47E-06	9.20E-07	1.27E-05
Exposure-upper	Coking plant area	3.10E+01	1.51E-04	1.08E-04	1.55E-04	9.84E-06	6.28E-04
Exposure-upper	Exposed residential area	1.80E+01	8.60E-05	5.87E-05	7.99E-05	8.78E-06	3.24E-04
Exposure-upper	Control area	3.80E+01	1.53E-05	1.03E-05	1.11E-05	4.14E-06	5.70E-05
